# Fluorescent antibiotics, vomocytosis, vaccine candidates and the inflammasome

**DOI:** 10.1002/cti2.1083

**Published:** 2019-11-01

**Authors:** Rachel J Lundie, Karla J Helbig, Jaclyn S Pearson, Kirsten A Fairfax

**Affiliations:** ^1^ The Walter and Eliza Hall Institute of Medical Research Parkville, Melbourne VIC 3052 Australia; ^2^ Department of Physiology, Anatomy and Microbiology School of Life Sciences La Trobe University Bundoora VIC 3086 Australia; ^3^ Centre for Innate Immunity and Infectious Diseases Hudson Institute of Medical Research Clayton VIC 3168 Australia; ^4^ Department of Medical Biology The University of Melbourne Parkville VIC 3010 Australia; ^5^ School of Medicine Menzies Research Institute Tasmania University of Tasmania Hobart TAS 7000 Australia

## Abstract

This article summarises recent advances reported at the 9th Lorne Infection and Immunity Conference. This exciting conference hosted speakers in the fields of innate and adaptive responses to infection including host–pathogen interactions as well as novel strategies for the detection, control and treatment of infectious diseases such as fluorescent antibiotics and vaccine development. Host–pathogen studies focused on a broad range of pathogens including malaria, CMV, influenza, dengue and Zika viruses, listeria and tuberculosis.

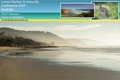

## Introduction

**Figure 1:**
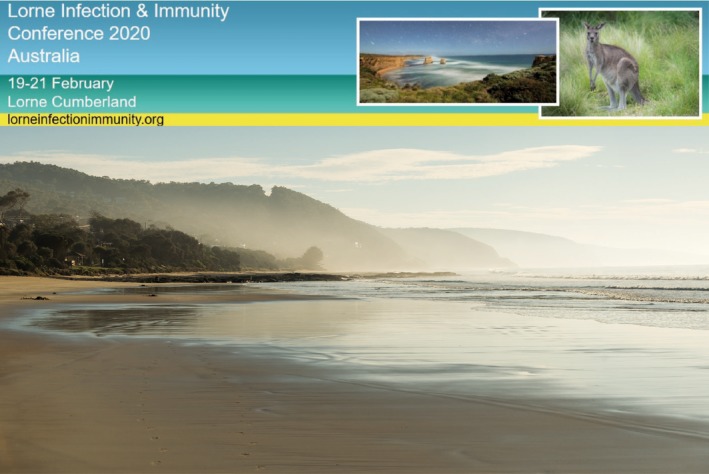
The beautiful beach at Lorne where the conference is held each year.

Scientists gathered on the 20–22 February 2019 for a fantastic multidisciplinary international meeting focusing on Infection and Immunity in the beautiful surrounds of Lorne, Australia (see Figure 1). We heard from experts working on innate and adaptive immune responses to infection, host–pathogen interactions and strategies for detecting infectious disease, as well as novel vaccination strategies and therapeutic avenues. Here, we summarise some of the main concepts discussed at the meeting, including unpublished data, and progress towards development of new therapeutic approaches for the control of infectious diseases.

### The leading clinical edge

Several presentations described ongoing clinical work to develop new tools or novel ways to use old tools for disease control in the Infection and Immunity in Translation session. Tuberculosis is the leading cause of death from infection worldwide, with an estimated 1.6 million deaths in 2017. The use of the attenuated Bacille Calmette–Guerin (BCG) vaccine gives variable protection against disease, but does not prevent infection. International invited keynote speaker Dr Robert Seder, a group leader from the National Institute of Allergy and Infectious Diseases, showed that delivering a BCG vaccine in a highly pathogenic nonhuman primate model using intravenous immunisation prevented infection and disease in the majority of animals compared to minimal protection against disease following an intradermal or aerosol route of delivery. After intravenous delivery of BCG vaccine, antigen‐specific T cells were observed to accumulate in the lung tissue, surpassing effects observed by intradermal or aerosol challenge. This work builds on initial work published more than 50 years ago and recent work by Michael Dennis’ group comparing intradermal, intratracheal and intravenous vaccination routes for BCG^1^, ^2^ and Dr Seder’s work on the intravenous administration of *Plasmodium falciparum* sporozoites (PfSPZ) as a vaccination strategy to induce sterile protection against malaria.^3^, ^4^

Of growing concern is the emergence of antibiotic‐resistant bacteria, with the World Health Organization listing antimicrobial resistance in its list of ‘Ten threats to global health in 2019’.^5^ Dr Mark Blaskovich, a senior research chemist at the University of Queensland, gave an elegant short presentation on using fluorescent antibiotics in diagnosis and studies of resistance, toxicity and mechanism of action. His team has been developing fluorescent antibiotics via synthetic conjugation of small fluorescent moieties such as NBD (Cy5, 7‐nitrobenz‐2‐oxa‐1,3‐dinzol‐4‐yl) or dimethylaminocoumarin‐4‐acetate to the antibiotic core in a site that does not interfere with antibiotic function, such as vancomycin‐NBD.^6^ Collaborators interested in applying these fluorescent antibiotics to the clinic are encouraged to contact Mark (https://imb.uq.edu.au/profile/929/mark-blaskovich).

The failure of antivirals to control cytomegalovirus (CMV) infection in the context of bone marrow transplants was discussed in a short presentation by Associate Professor Barry Slobednam, a group leader at the University of Sydney. The majority of bone marrow transplants have either a donor or a recipient who is CMV+. The work presented aims to predict CMV reactivation using both whole CMV genome sequencing strategies to define the emergence of anti‐CMV drug resistance mutations, and mass CyTOF (cytometry time‐of‐flight) to study 36 cell surface markers on PBMCs at the single‐cell level.^7^ The goal is to determine novel biomarkers for viral reactivation or severe graft‐versus‐host disease onset, which could be used to provide earlier intervention points for greater efficacy of existing treatment.

### Viral infections and host responses

Many prominent research themes involving viral pathogens and their host interactions were presented at the meeting. Dr Sarah Londrigan, a senior research officer at The Doherty Institute, gave a short talk in the Innate Immunity session describing novel research into differences between macrophages and epithelial cells in clearance of seasonal influenza strains, showing that human alveolar macrophages demonstrate a late‐stage block in virion release. Dr Londrigan’s work is now investigating if the abortive block in influenza egress from macrophages is associated with defective plasma membrane localisation of viral proteins. Interestingly, obesity has been identified by the Centre for Disease Control and Protection as a risk factor for enhanced viral severity to influenza,^8^ which was highlighted by our national invited speaker in the Viruses and their Hosts session, Dr Kirsty Short, an Australian Research Fellow from the University of Queensland. Dr Short’s research has found that obese mice with influenza infection present no differences in viral titres throughout multiple organs, but become hypoxic with significantly higher left ventricular mass coinciding with increases in inflammation scores and serum creatine kinase‐MB. Given the rise of obesity in populations worldwide, this work represents great insight into future clinical patient management during influenza outbreaks.

Arboviral‐driven infection remains a significant problem worldwide, and work presented in the Viruses and their Hosts session revealed that both novel treatment avenues and reduction in the spread of these viruses are being tackled from multiple fronts. A short presentation by Dr Prasad Paradkar, a group leader at CSIRO, detailed development of virus‐resistant engineered mosquitoes that do not transmit saliva‐mediated virus to mammalian hosts. The strategies presented, including siRNA targeting viral genomes or single‐chain neutralising antibodies,^9^ mediated almost a complete loss of detectable Zika or dengue virus replication in the midgut and whole body of mosquitoes homozygous for the novel traits. Work presented by the international invited speaker in this session, Professor Shee‐Mei Lok, from the DUKE‐NUS Medical School in Singapore, described the first structure of the Zika virus capsid protein, demonstrating a triangular network of capsid dimers, knowledge that will be paramount in development of successful Zika virus vaccine candidates.^10^

### The latest on the inflammasome

The international keynote speaker in the Host‐Pathogens Interactions II session, Dr Kim Newton, a senior scientist from Genentech in California, presented on why immunotherapies for inflammatory diseases aimed at inhibiting inflammatory cytokines are ineffective. Her theory is that cell death processes may be responsible and that caspase‐8 inhibition, possibly by gut pathogens, allows other inflammatory processes, such as inflammasome activation involving RIPK1, to be promoted. Conversely, Dr James Vince and Dr Kate Lawlor from the Walter and Eliza Hall Institute for Medical Research (WEHI) and the Hudson Institute, respectively, presented short talks in the Innate Immunity session exploring the mechanisms by which Bax/Bak promotes loss of the inhibitors of apoptosis, which in turn activate caspase‐8 and induce IL‐1β production.^11^ With research into modulating the inflammasome a hot topic at the moment, in addition to the invited speakers, there were several impressive early career presentations, including Dr Rebecca Coll’s (University of Queensland) PerkinElmer prize‐winning presentation on the NLR pyrin domain containing 3 inflammasome inhibitor, MCC950.

### Microbial pathogens and host mechanisms of defense

Microbial pathogens have evolved over millennia to escape and/or manipulate host responses to survive, persist and cause disease. Globally, researchers are on a mission to decipher the underlying mechanisms of host–pathogen interaction to gain a more complete understanding of innate immunity to various organisms and autoimmune disease processes.

Invited international plenary speaker, Professor Pascale Cossart from the Pasteur Institute, delivered a comprehensive overview of her studies on *Listeria* 12 in the Hartland Oration session. In addition to the well‐described listeriolysin O,^13^, ^14^, ^15^ Professor Cossart described listeriolysin S as a newly described bacteriocin expressed in the intestine that specifically targets commensal organisms of the genera *Alloprevotella* for depletion to promote the spread of epidemic *Listeria* strains.^16^ Her group has further evidence of another *Listeria* bacteriocin (unpublished) that specifically targets another gut bacterium, *Prevotella copri*, that modulates infection and potentially facilitates persistence of infection – *watch this space!*

Hosts have learnt many tricks to control microbial pathogens, as we learnt from our opening invited international speaker, Professor Robin May from the University of Birmingham, whose group has coined the term *vomocytosis* to describe nonlytic extrusion of pathogens by macrophages. Professor May used the invasive fungal pathogen *Cryptococcus* to demonstrate that this phenomenon is regulated by extracellular signal‐regulated kinase 5.^17^ The underlying principle of *vomocytosis* is that spitting the pathogen out of the cell at earlier time points limits dissemination and thus controls the pathogen. Interestingly, viral co‐infection (e.g. HIV or measles) with *Cryptococcus* increases *vomocytosis*, a process driven by interferon‐α.^18^ There were many outstanding speakers in the field of microbial pathogens, including early career researcher Georgina Pollock, a PhD student at The Hudson Institute, who presented her work on the characterisation of bacterial effector kinases.

### Breakthrough research in the fight against malaria

Structural biology approaches using revolutionary cryo‐electron microscopy have facilitated breakthroughs in understanding how *Plasmodium* invades human red blood cells. Dr Wilson Wong, a postdoctoral researcher from WEHI, gave a short presentation on the characterisation of the structure of the leading blood‐stage malaria vaccine candidate, the *P. falciparum* Rh5‐CyRPA‐Ripr protein complex, which binds to the receptor basigin on the erythrocyte surface.^19^ Key findings included identification of CyRPA as a critical mediator of complex assembly and the discovery that both Rh5 and Ripr are positioned parallel to the erythrocyte before membrane insertion. Meanwhile, invited national speaker Associate Professor Wai‐Hong Tham, a group leader at WEHI, revealed a detailed view of the interaction surfaces between *Plasmodium vivax* reticulocyte‐binding protein 2b (PvRBP2b) and transferrin receptor 1 (TfR1).^20^ Notably, PvRBP2b residues critical for complex formation with TfR1 are conserved across genetically diverse *P. vivax* species, providing strong rationale for including these pan‐reactive epitopes as future *P. vivax* vaccine candidates.

Malaria researchers are also making rapid advancements in drug discovery, with invited international speaker Professor Elizabeth Winzeler from the University of California providing an inspiring update on the Malaria Drug Accelerator (MalDA) initiative. MalDA is a collaboration among 13 scientific laboratories developing antimalarial drugs. Professor Winzeler’s laboratory uses multiple complementary approaches, including whole‐genome sequencing, phenotypic screening and functional studies of host–parasite interactions, to discover new drug targets and their mechanism of action. Researchers who have identified a novel drug target and would like to work in partnership with the programme are encouraged to contact Professor Winzeler (https://winzeler.ucsd.edu).

Exciting progress in translational malaria research was further presented by the national invited speaker Professor James McCarthy from QIMR Berghofer Medical Research Institute, who has pioneered experimental human malaria infection models to study immunity to malaria, test new antimalarial drugs and increase understanding of the *in vivo* biology of ‘bespoke parasites’ in controlled conditions. The most promising data were from a pilot study of an FDA‐approved live attenuated blood‐stage vaccine candidate, composed of genetically attenuated *P. falciparum* 21 (ANZCTR registration number: ACTRN12617000824369). Healthy human volunteers infected with a high dose (3 million) of the live attenuated vaccine generated robust malaria‐specific antibody responses, similar to levels detected in naturally exposed populations. Future studies aim to extend these findings by challenging vaccinated volunteers with wild‐type parasites to determine the efficacy of this novel vaccination strategy. Outstanding early career presentations in the field of clinical malarial research included PhD student Madeline Dans (Burnet Institute/Deakin University) and Dr Liriye Kurtovic (Burnet Institute), who were awarded prizes for their interesting work into phenotypic screening and vaccine development, respectively.

## Conclusion

Understanding host–pathogen interactions through multidisciplinary approaches has been key to recent successes in generating treatments or vaccines to combat some of the key pathogens threatening the health of humans worldwide. Bringing together a range of immunologists, infectious and inflammatory disease specialists, and microbiologists to create collaborations and discuss ideas is what makes this meeting unique and provides new and cross‐disciplinary approaches to research. We look forward to February 2020 for the 10th Lorne Infection and Immunity Conference (://www.lorneinfectionimmunity.org/).

## Conflict of Interest

The authors have no conflicts of interest to disclose.
